# Smart Data Placement Using Storage-as-a-Service Model for Big Data Pipelines

**DOI:** 10.3390/s23020564

**Published:** 2023-01-04

**Authors:** Akif Quddus Khan, Nikolay Nikolov, Mihhail Matskin, Radu Prodan, Dumitru Roman, Bekir Sahin, Christoph Bussler, Ahmet Soylu

**Affiliations:** 1 Department of Computer Science, Norwegian University of Science and Technology—NTNU, 2815 Gjøvik, Norway; 2 SINTEF Digital, SINTEF AS, 0373 Oslo, Norway; 3 Department of Computer Science, KTH Royal Institute of Technology, 114 28 Stockholm, Sweden; 4 Department of Information Technology, University of Klagenfurt, 9020 Klagenfurt, Austria; 5 Logistics Management, National University of Science and Technology, 111 Sohar, Oman; 6 Robert Bosch LLC, Sunnyvale, CA 94085, USA; 7 Department of Computer Science, OsloMet—Oslo Metropolitan University, 0167 Oslo, Norway

**Keywords:** storage-as-a-service, big data pipelines, data locality, data placement strategies, software containers

## Abstract

Big data pipelines are developed to process data characterized by one or more of the three big data features, commonly known as the three Vs (volume, velocity, and variety), through a series of steps (e.g., extract, transform, and move), making the ground work for the use of advanced analytics and ML/AI techniques. Computing continuum (i.e., cloud/fog/edge) allows access to virtually infinite amount of resources, where data pipelines could be executed at scale; however, the implementation of data pipelines on the continuum is a complex task that needs to take computing resources, data transmission channels, triggers, data transfer methods, integration of message queues, etc., into account. The task becomes even more challenging when data storage is considered as part of the data pipelines. Local storage is expensive, hard to maintain, and comes with several challenges (e.g., data availability, data security, and backup). The use of cloud storage, i.e., storage-as-a-service (StaaS), instead of local storage has the potential of providing more flexibility in terms of scalability, fault tolerance, and availability. In this article, we propose a generic approach to integrate StaaS with data pipelines, i.e., computation on an on-premise server or on a specific cloud, but integration with StaaS, and develop a ranking method for available storage options based on five key parameters: cost, proximity, network performance, server-side encryption, and user weights/preferences. The evaluation carried out demonstrates the effectiveness of the proposed approach in terms of data transfer performance, utility of the individual parameters, and feasibility of dynamic selection of a storage option based on four primary user scenarios.

## 1. Introduction

The exponential growth of digital data sources has the potential to transform every aspect of society and human life. To achieve this impact, however, data must be processed swiftly for extracting insights through advanced analytics and machine learning (ML) or artificial intelligence (AI) techniques and driving decision-making processes. Big data pipelines are designed to support one or more of the three big data features, commonly known as the three Vs (volume, velocity, and variety), while processing data through a series of data processing steps (e.g., extract, transform, and move) [1]. Therefore, in order to execute data pipelines at scale, it is critical to develop innovative methods for efficiently using the available distributed computing infrastructure and resources, i.e., the computing continuum encompassing cloud/fog/edge resources and services [2]. The computing continuum allows access to a virtually infinite amount of resources, where data pipelines could be executed at scale; however, the implementation of data pipelines on the computing continuum is a complex task that needs to take computing resources, data transmission channels, triggers, data transfer methods, the integration of message queues, etc., into account. This process becomes even more complex if data pipelines are coupled with data storage, e.g., distributed file systems, which come with additional challenges such as data maintenance, security, scalability, etc. [3]. Local storage is expensive, hard to maintain, and comes with several challenges (e.g., data availability, data security, and backup). Storing big data on traditional physical storage on-premise is troublesome, as hard disk drives (HDDs) regularly fail, and specific data protection measures, e.g., RAID, are not always efficient [3]. The use of cloud storage, i.e., storage-as-a-service (StaaS) [4], instead of local storage, has the potential to provide more flexibility in terms of scalability, fault tolerance, and availability. Cloud storage systems (e.g., Amazon S3, Elastic Block Store, or EBS, Azure Blob Storage, and Google Cloud Storage) offer very large storage with high fault tolerance, addressing several big-data-related storage concerns [5].

**Figure 1:**
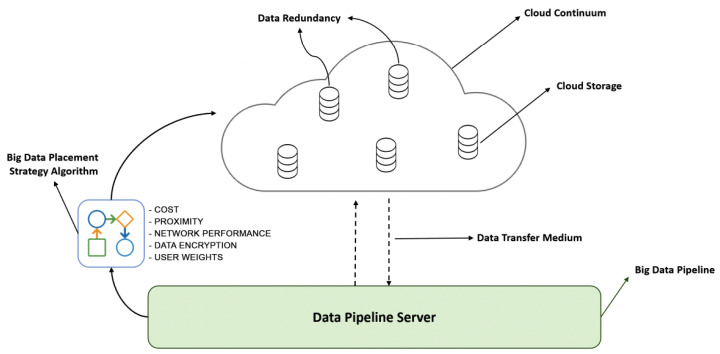
Big data pipelines with StaaS and smart data placement.

Moving to and hosting big data on the cloud shifts the extra overhead of data redundancy, backup, scalability, security, etc., to the cloud service provider [6]; however, it could be expensive given the scale of data volume [5]. Therefore, principles and algorithms, including the spatiotemporal patterns of data consumption, need to be created to establish the data’s analytical value and its preservation by balancing the expense of storage and data transfer with the quick accumulation of big data [7]. In addition, the pace of big data necessitates the storage systems to scale up fast, which is challenging to achieve with standard storage systems. That is why it is important to either select a suitable storage facility before data placement or move the compute step closer to the data during data processing. In this respect, the objective of this article is to demonstrate that the integration of StaaS with big data pipelines is a promising direction. This necessitates a one-of-a-kind solution for data pipeline design and a method for real-time data placement that takes into account constraints such as unknown data volumes, availability, location, data security, etc. To this end, we first propose an approach to realize big data pipelines with hybrid infrastructure, i.e., computation on an on-premise server or on a specific cloud, but integration with StaaS; and secondly develop a ranking method for predeployment stage to find the most suitable storage facility, dynamically, based on the user’s requirements, including cost, proximity, network performance, server-side encryption, and user weights (i.e., preferences) [8,9]. Figure 1 demonstrates the solution approach on a multicloud environment, i.e., cloud continuum, where storage and computation could be distributed over multiple cloud providers, and a cloud storage provider could be selected dynamically based on the aforementioned parameters. Several evaluations were carried out to demonstrate the effectiveness of the proposed approach in terms of data transfer performance, the utility of the individual parameters, and the feasibility of dynamic selection of a storage option based on four primary user scenarios, including scenarios covering temporary requirements, high variable workloads, high-security and low-scale solutions, and dormant workflows.

The rest of the article is organized as follows: Section 2 sets the background, while Section 3 provides the related work. Section 4 presents the proposed approach and describes the ranking method used. Section 5 provides the evaluation of the approach, while Section 6 concludes the paper.

## 2. Background

Big data has quickly gained popularity because it enables the exploitation of data to discover hidden trends that provide important insights to drive corporate decisions and aid research. It has been successfully applied in a wide variety of fields, including marketing, social network analysis, healthcare, and finance (e.g., [10,11]). The widespread use of big data analytics stems from the idea that with big data, many real-world occurrences can be better understood and predicted. However, creating and deploying algorithms and tools that extract value from large amounts of data adds to the complexity and cost of the resources needed for producing complete big data solutions.

Cloud computing offers virtually unlimited resources (e.g., storage and computation) on an on-demand basis, typically using a pay-as-you-go approach and giving users the opportunity to manage the cost. The cloud is a centralized system that stores data, runs application logic, and handles data and analytics duties while being located far away from end users and data sources. It offers vast computing capabilities, scalability, durability, and a cheap initial cost [12]. Yet, data-intensive applications requiring low-latency computing and substantial resources for complex processing are hard to realize with only distant cloud centers [13]. In this respect, the computing continuum offers the required elasticity in terms of resources needed and meets the latency requirements by extending cloud computing with fog (i.e., a set of nodes with a local network placed between the cloud and edge) and edge computing paradigms (i.e., a set of local nodes near a data source that process data independently). It enables low-latency data processing among others, such as reduced battery consumption, enhanced user experiences, location awareness, etc. [14], by pushing computing and storage services closer to the data sources [15,16] and encompasses a diverse set of capabilities and services that are readily linked by cloud-first networks and governed by complicated standards.

**Figure 2:**
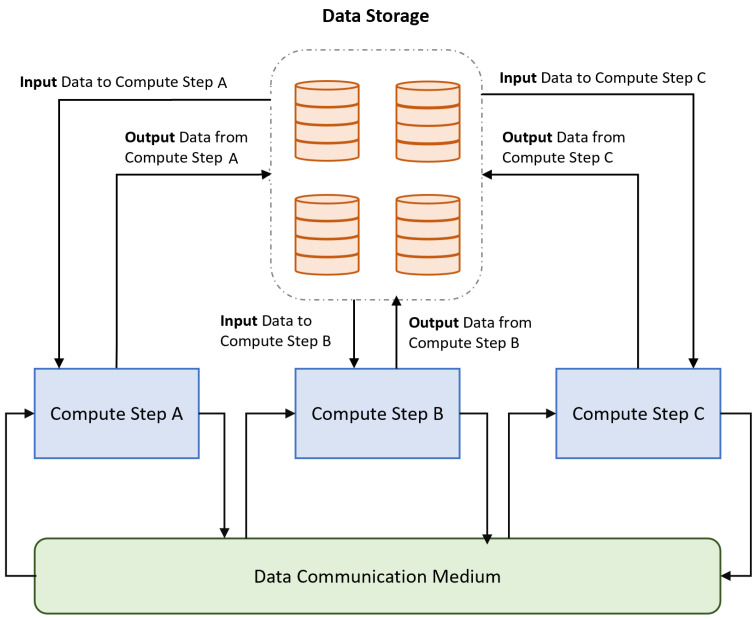
An example pipeline architecture.

A data pipeline is composed of atomic units, called steps, applied to data (e.g., cleaning, enriching, and transforming) [17,18]. Using a basic generalization, each step receives data as input from a data store and processes it before pushing the outputs back to a data storage. Processed data travel over the computer network using the data transmission medium. The inputs and outputs of a step are coupled to such channels. The results of one stage must be used as input for the following step in the pipeline. Control messages, such as notifications and triggers for the other steps, are also passed through the communication medium. Some common examples include KubeMQ, ZeroMQ, etc. An example data pipeline architecture is depicted in Figure 2. An example pipeline could include processing data from multiple streams that are gathered and stored by a cloud storage provider. A data transfer medium is set up between the cloud storage and the data pipeline server. For data transfer, the HTTP or FTP protocols could be used. For this experiment, we used the standard HTTP protocol. A message queue is implemented on the data pipeline server for interstep communication. The integration can either be performed with single cloud storage or multiple cloud storage. For example, it is possible to store data collected from various streams in the storage facility offered by a storage provider, but the data after processing could be stored with another cloud storage provider.

StaaS is the practice of storing data on public cloud storage facilities with varying differences in the data model, consistency, semantics, transaction support, and pricing model [4]. Transferring large amounts of data over long distances will introduce latency issues. In this respect, in a distributed system, data locality is critical for data processing performance. It refers to bringing computing closer to the data, which is frequently less expensive and faster than moving data closer to the computing. Network traffic and the latency associated with data transmission between computers may have an impact on the overall cost and performance of big data processing due to the large volume of data. As a consequence, the capability of the work to be distributed in a way that minimizes data transfers (data locality) has been explored and implemented in a number of studies [17]. Even for centralized deployment systems (such as cloud deployments), data locality has been shown to be effective in reducing costs and execution times [19,20]. The locality of data is just one factor that might be considered when scheduling operations. Other concerns, such as load distribution and heterogeneity of available resources among nodes, must be balanced against data locality in order to perform operations efficiently [21].

## 3. Related Work

The scientific community has extensively acknowledged the necessity of using cloud computing to execute scientific workflows/pipelines [22]. Many studies investigated and demonstrated the viability of employing cloud computing for deploying big data pipelines in terms of both cost [23] and performance [24].

Abouelhoda et al. [25] proposed Tavaxy, a system that enables seamless integration of the Taverna system with Galaxy processes based on hierarchical workflows and workflow patterns. Wang et al. [26] presented early results and experiences in enabling interaction between Kepler SWFMS and the EC2 cloud. Antonio et al. [27] analyzed hybrid multicloud storage systems and different data transfer techniques in general. These approaches discuss the benefits and possibilities of deploying big data pipelines on cloud infrastructure; however, they do not discuss the possibility of hybrid big data pipelines in a multicloud environment. Zhang et al. [28] proposed BerryStore, a distributed object storage system suited for the storage of huge quantities of small files. In a large Web application, file sharing generates a large number of requests. BerryStore is built to manage these requests. The essential insight is that extraneous disk operations should be avoided when reading metadata. The proposed mechanism does not provide support for integration with the big data pipelines. Yuan et al. [29] studied the unique characteristics of scientific cloud operations and proposed a clustering data placement strategy capable of dynamically moving application data across data centers based on dependencies. Simulations on their cloud workflow system, SwinDeW-C, showed that their data placement strategy may significantly reduce data traffic during the execution of the process. The suggested system is heavily platform-dependent since it only works for Hadoop. There are several other data placement techniques developed for Hadoop [30,31,32].

Er-dun et al. [33] addressed the issues connected with scientific workflows in the cloud computing environment, specifically the load balancing of data centers. After examining the storage capacity of data centers, data transit patterns, and data center loads, they proposed a workable data placement strategy using a genetic algorithm. In comparison to other data placement strategies, the genetic-algorithm-based data placement methodology performed well in terms of data center load balancing and data movement volume. While the proposed technique is effective, it does not address the functional requirements of the owners or developers of big data pipelines. It is limited to the number of datasets and the number of movements. Halimi et al. [34] proposed a QoS-focused approach for storage service allocation. The proposed hybrid multiobjective water cycle and gray wolf optimizer (MWG) takes into account a variety of QoS objectives (such as energy, processing time, transmission time, and load balancing) in both the fog and cloud layers, which have not received much attention in the past.

Ilieva et al. [35] proposed a method for choosing cloud storage as a fuzzy multicriteria problem. They considered different factors, such as product features, functionalities, customer support, and security options for evaluating cloud technologies, and linguistic phrases, relative weights, and crisp values were converted into triangular fuzzy numbers before being used in the multicriteria analysis. Liu et al. [36] addressed security and availability issues during the data placement process by presenting an architecture named High Available Cloud Storage Gateway (HASG). In their design, a data file is divided into implicit and redundant blocks by a file fragment algorithm. Each block of data is stored in several chunks and stored on different designated cloud servers. These chunks, at the time of retrieval, can be used to reconstruct the original file. Oki et al. [37] proposed cloud storage selection models for cloud storage to satisfy data availability requirements. They implemented a prototype for cloud storage and demonstrated new models. All these approaches lack the ability to address a complete set of user requirements. For example, if an approach is developed to achieve high data availability, it does not consider the data security factors and their impact on the availability, and vice versa.

Xiahou et al. [38] presented data placement and retrieval methods for the HDFS storage system. They proposed AHP-backward cloud generation-algorithm-based cloud service selection strategy for data centers. They proposed a cloud storage framework that analyzes an HDFS storage system, an architecture over multiple data centers, and a selection strategy based on a replica layout mechanism and data consistency maintenance strategies. Regarding the former, Zhao et al. [39] also presented data placement and retrieval methods for an HDFS storage system. They studied the data replica selection and replication strategies by taking into consideration bandwidth, the performance of peers, and the replica’s history request. An algorithm was developed for HDFS, which reduces the running time and balances the node load.

These approaches discuss the benefits and possibilities of deploying big data pipelines on cloud infrastructure; however, they do not discuss the possibility of hybrid big data pipelines. In addition to that, some of them only focus on one cloud provider; the possibility of hybrid big data pipeline implementation with multicloud platforms has not been discussed. The approach proposed in this article incorporates multiple cloud storage providers, focuses on big data pipelines, allows dynamic selection of storage providers, and is platform-independent.

## 4. Smart Data Placement

**Figure 3:**
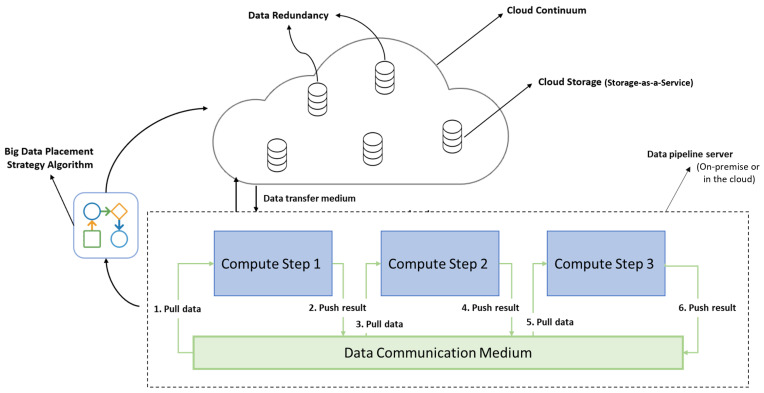
Smart data placement using storage-as-a-service approach.

The proposed approach is shown in Figure 3, where the compute steps of a data pipeline are encapsulated in software containers, as suggested by [17], and deployed on a server labeled as the data pipeline server. The data pipeline server could either be on-premise or in the cloud. In the context of this work, the data pipeline server is deployed on-premise. A communication medium is setup for interstep communication, for example, pulling and pushing information about different pipeline events (trigger events) and execution results. The local storage, in this proposed approach, is replaced with hybrid cloud storage as a service. To reduce the cost of network egress in the case of on-premise computation servers, the interstep data storage concept is also introduced. We find the most suitable storage facility at the predeployment stage to store data from the data pipeline server using a ranking method that takes a set of parameters into account.

**Figure 4:**
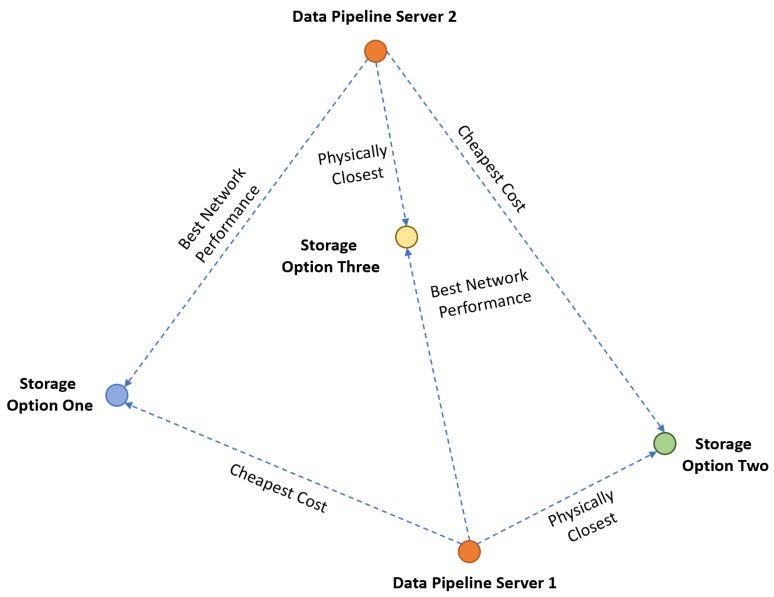
Relation between cost, network performance, and proximity.

There are several parameters that affect the choice of cloud storage, such as the cost of storage space, security, performance, and location. Cloud providers come with a variety of storage, computation, and network resources; therefore, in order to make an informed choice, multiple parameters need to be taken into account. In this study, five primary parameters were selected for the evaluation of different cloud storage providers, which are (1) cost, (2) proximity, (3) network performance, (4) impact of server-side encryption, and (5) user weights/preferences. Cost is one of the major reasons for application providers to move their services to the cloud [4] and is a complex construct. Proximity, or distance in this context, is selected since it has a direct impact on communication latency. Proximity can provide an indication of which facility is likely to provide the best data transfer performance; however, the actual network performance tests (bandwidth, throughput, latency, etc.) can provide further information, while noting that the proximity is a more permanent characteristic than the network’s performance. The fourth parameter is the impact of server-side encryption for the sake of data security, which is a strong concern for the adoption of cloud solutions [40]. Finally, user preferences are used to allow decision-makers or users to communicate their preferences. These parameters are interdependent on each other; therefore, there is a possibility that the cloud service provider that fulfills security requirements does not have the best network performance, or that the one closest to the data pipeline server is more expensive. This scenario is visually represented in Figure 4. The focus of the proposed method is mainly on data locality, that is, smart data placement to achieve maximum performance output; however, different locations with different cloud storage providers have different costs. In addition to that, all data centers have different network infrastructures, so network performance can vary between different data centers. The challenge is to decide which criteria are best suited to the situation and user requirements. In the following, we discuss the first four parameters in detail, and the last one is discussed as part of the ranking method.

### 4.1. Cost Model

To estimate cost, it is important to collect and then normalize data about the offered services and their pricing structures [4]. For example, to estimate the total cost, it is required to take into account not just the volume of data to be stored but also the cost of network usage in terms of the concrete number of READ and WRITE operations as well as the amount of data transferred. Moreover, each cloud storage provider has multiple options for storage services. For example, Microsoft Azure (Azure) has three different tiers, such as the hot tier, the cool tier, and the archive tier. Google Cloud Platform (GCP), on the other hand, has exactly the same structure for storage options, but with different names (standard, coldline, and archive). Amazon Web Services (AWS) follows the same pattern but with different names. Similarly, network usage is further classified into egress (i.e., outbound traffic from a network) and ingress (i.e., traffic that originates outside the network and enters the network). When it comes to the selection of a storage facility, the user is given the option of multiple regions and availability zones. The term "availability zone" refers to a specific geographic area in which a single data center is located. For data normalization, it is important to select common services from each of the providers. For example, each service provider has several storage tiers and different pricing models for each tier.

We calculate the cost based on four variables using one tier from each storage provider with similar characteristics: the storage space itself, the out bandwidth, and the number of READ and WRITE operations. When it comes to the cost of cloud storage, it is not just about the price per gigabyte (GB). Additionally, there are fees involved with moving data between the cloud and on-premise systems. In many services, there are two costs: one per-gigabyte cost each time servers in different domains communicate with each other, and another per-gigabyte cost to transfer data over the internet. The user requirements are used as an input set to estimate the cost. This includes the total amount of storage required in GB. The unit GB is selected instead of TB to provide more flexibility to the user. The second parameter specifies the required bandwidth in GB. The third and fourth parameters specify the number of write and read operations, respectively. Another input set is the normalized cost data for each provider, similarly in terms of cost for storage per gigabyte, bandwidth in and out per gigabyte, and read and write operations per a specific number.

### 4.2. Proximity

Big data processing requires pooling the resources of multiple machines in a distributed system while hiding the complexities of the distributed resources behind a single interface. This requires data to move across machines, which in turn leads to latency, which is a critical factor for many use cases requiring low latency. Data locality [41] is a viable approach to address the bottleneck problem of data having to travel through slow networks. Data do not need to circulate between a VM and remote hosts all the time as necessary if datasets are available locally. It also keeps the I/O operations within a single physical node. Thus, data locality allows for avoiding network stack processing overhead, resulting in significantly lower latency because much less data are transferred via the network.

Physically closest storage data facilities, although not always, have a higher possibility of resulting in better data transfer performance and could be one of the considerations when deciding on where to deploy the data (also in terms of security, etc., considerations). We calculate the physical distance between the data pipeline server and the data centers based on the longitude and latitude information and using IP ranges provided by the cloud service providers and GeoIP (not the elastic IP assigned for the user’s personal use). For example, for the details of AWS storage facilities in real time from AWS IP Ranges, for Azure, the list of instances is downloaded in real time from Azure IP Ranges, and the IP ranges for the GCP are downloaded from GStatic. Based on the IP, one does not obtain the exact pinpoint location of the storage server, but based on this information, one obtains the proximity. Note that the physical distance has two elements to it: one is the great circle distance, and then one is the actual network fiber distance. Therefore, one should not assume that a network connection uses the shortest physical connection, and one also needs to take into account the fact that routes might dynamically change, introducing spikes as the fiber distance could be longer or shorter. Therefore, an alternative/complementary approach is to dynamically measure the ping round trip between the data pipeline server and the cloud storage providers.

### 4.3. Network Performance

Network performance and the quality of network services play a crucial role in the efficiency of operations and the end-user experience of the applications deployed in a distributed computing environment. Due to the variety of network types and architectures, a wide range of performance evaluation methods are available (e.g., [42]). In order to assess the overall quality of a data center’s network, a collection of network statistics is examined and evaluated. It is a quantitative technique that assesses and specifies the degree of performance of a network under consideration. In this context, in general terms, bandwidth, throughput, and latency, among others (e.g., jitter, loss, error, etc.), are the key network performance metrics. Latency refers to the time required to send a data packet from one point to another in a network and is mostly measured in terms of the round-trip time (RTT). Bandwidth refers to the amount of data that could be transferred within a specific time period through a network (i.e., network capacity) and is usually measured in terms of bits per second and megabits per second, while throughput specifies the amount of data successfully transferred through a network over a specified period of time.

There are many different techniques to assess its performance since each network is unique in its nature and architecture. In order to assess the performance of the network in real time, we deployed a sample data pipeline and measured the throughput. A dataset with smaller data chunks and a pipeline with a large number of operations are selected to measure the network connection time for each operation. The pipeline not only executes READ operations; it also executes WRITE operations. Regarding the relevance of different metrics, for really small datasets, such as for REST-based APIs for transactional systems, latency is more relevant, since in a transactional system it is desired that individual service invocation should return as fast as possible. However, for large datasets, the latency is less relevant, but the throughput is highly relevant, since the higher the throughput, the shorter the time it takes for the whole amount of data to be transferred. In architectural terms, one would avoid accessing data remotely but instead move the computation near the data. For example, this could be achieved by having the same pipeline step available in different regions, while the data are in different regions, and accessing the data locally in the same region by the pipeline step (assuming the data passed between steps are small compared to the set of data accessed within a step). Therefore, data access by steps and data passed between steps are the two main dimensions. Ideally, a single step only accesses a dataset in one location to avoid remote access, but the nature and architecture of the pipelines could vary.

### 4.4. Server-Side Encryption

Security is another vital consideration for application providers considering adopting cloud storage solutions [36]. Data security in the computing continuum is a fuzzy notion with complex legislation, security, and privacy considerations, which require technical and managerial solutions and location-awareness in a multicloud environment [4]. In this work, our focus is on data encryption, in particular, server-side encryption (as opposed to client-side encryption), so as to transfer the overhead to the provider side as much as possible. It is the encryption of data at their destination by the application or service that receives them. Cloud storage providers encrypt the data at the object level as they write them to disks in their data centers and decrypt the data for the user when they are accessed. As long as the request is authenticated and access permissions are properly defined, there is no visible difference in the way to access encrypted or unencrypted objects. For example, if objects are shared using a preassigned URL, that URL works the same way for both encrypted and unencrypted objects. Server-side encryption is offered by most of the providers, but implementation details differ, particularly for the management of keys.

In order to assess the impact of encryption on the performance of a data pipeline, a simple two-step data pipeline is developed. A dataset is stored in cloud storage and encrypted using server-side encryption. The first step downloads the data from the server and stores them temporarily in local storage. The second step reads the data from the local storage and uploads them back to the cloud storage. We used relatively bigger data chunks to correctly observe the impact of decryption on the performance while retrieving the data. Two separate storage buckets are deployed in the same physical region for each storage provider. Server-side encryption is enabled for one bucket, whereas in the other bucket, data are stored without any encryption. Although a similar impact on the performance is expected for different providers, it can still vary between providers and their regions due to differences in implementation. It is also possible that the test pipeline could be impacted by the temporary slowdowns unrelated to the encryption.

### 4.5. Ranking Method

In order to be able to rank the cloud providers, we used multicriteria decision analysis (MCDA), which is an operation research discipline that deals with decision-making using multiple conflicting criteria (e.g., [43,44]). MCDA problems include a goal, opinions, or preferences of decision-makers or experts, decision alternatives, evaluation criteria, and outcomes. In this study, we used the VIKOR method [45], which is selected based on the generalized framework developed by Jankowski et al. [46]. The framework analyzed a collection of 56 available MCDA methods, and a hierarchical set of method features and a rule base were created as a result. It is an innovative tool for choosing the MCDA approach that is most appropriate for the decision problem. The acronym VIKOR stands for VlseKriterijumska Optimizacija I Kompromisno Resenje (Multicriteria Optimization and Compromise Solution), which was developed by a Serbian researcher in 1998 [47]. VIKOR provides a compromise solution [48] and, in the literature, the VIKOR algorithm is highly preferred not only in operations research and decision-making problems [49,50] but also in big data, information technology, and knowledge management fields. There are many different VIKOR applications and solutions regarding big data, such as selection criteria of the big data analysts [51], big data management in healthcare [52], big data solutions for dairy supply chains [53], augmented reality [53], mining big data [54], and so on.

**Figure 5:**
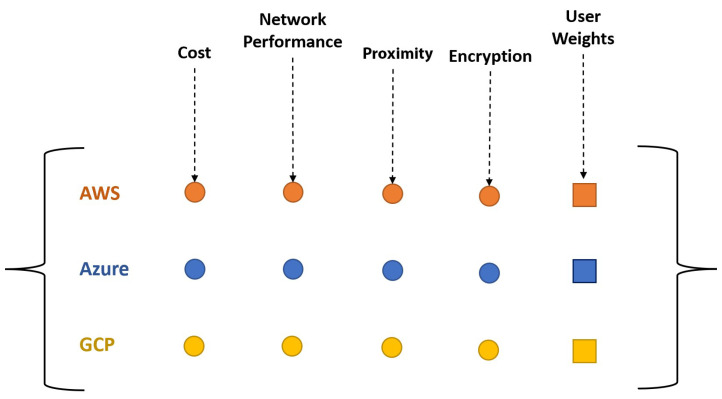
Evaluation matrix used for ranking cloud storage providers.

For this very evaluation model, the four different parameters are used in addition to the user weights. These parameters are as follows: cost (i.e., based on storage, bandwidth, and READ and WRITE operations—see [9]), proximity (i.e., using IP ranges provided by the cloud service providers and GeoIP), network performance (i.e., throughput), and the impact of server-side encryption (i.e., performance). Figure 5 shows the evaluation matrix for the proposed method. Values for the parameters are calculated using the independent software tools we developed, as described earlier. The last column shows the weights set by the user or the decision-maker. The evaluation matrix explained above is given as input to the MCDA VIKOR algorithm, and the output is the ranking of the cloud service providers. The VIKOR algorithm determines and achieves the compromise order (rank) for alternatives or options and compromise solution based on several predefined weights of criteria or parameters in complex decision-making problems. The steps of the VIKOR algorithm are provided below [49].

#### 4.5.1. Determine and Define the Alternatives and Criteria—Construct the Decision Matrix

As was mentioned before, VIKOR is an optimization technique where there exist several predetermined and defined alternatives (indicators, options, etc.) and criteria (parameters, requirements, etc.) for the decision-making problem, and it aims to find the best one among other alternatives considering the contributing criteria. Assume that the problem *F* has an alternative *i* with respect to the criterion *j*. The crisp or fuzzy data are needed to score the alternatives. In most cases, expert evaluation (subjective judgment), which commonly depends on the scales, is required to find the numerical values of alternatives $f_{ij}$. The characteristics of the criteria are also needed whether they are cost or benefit.

The decision problem in matrix form is shown in Equation (1):

(1) $$\begin{array}{c} F=\left[\begin{array}{ccccc} f_{11} & f_{12} & f_{13} & \cdots & f_{1n}\\ f_{21} & f_{22} & f_{23} & \cdots & f_{2n}\\ \vdots & \vdots & \vdots & \ddots & \vdots \\ f_{m1} & f_{m2} & f_{m3} & \cdots & f_{mn} \end{array}\right] \end{array}$$

where *m* and *n* represent the number of alternatives and criteria in the decision matrix, respectively, and in the following, indices *i* and *j* represent the relevant alternative or criterion in the matrix.

#### 4.5.2. Normalize the Decision Matrix

Normalization is the reorganization of the data by obtaining the normalized decision matrix (*R*) where its elements are $r_{ij}$. The following formulas in Equations (2) and (3) are used to find the $r_{ij}$ values.

(2) $$r_{ij}=\frac{f_{j}^{+}-f_{ij}}{f_{j}^{+}-f_{ij}^{-}}$$

where

(3) $$f_{j}^{+}=\max_{i}f_{ij}\;\mathrm{and}\;f_{j}^{-}=\min_{i}f_{ij}$$

#### 4.5.3. Obtain the Weighted Normalized Decision Matrix

In the third step, the weights of each criterion are multiplied by the corresponding $r_{ij}$ values (Equation (4)) to derive the weighted normalized decision matrix, as in Equation (5).

(4) $$v_{ij}=r_{ij}\cdot w_{ij}$$

(5) $$\begin{array}{c} V=\left[\begin{array}{ccccc} v_{11} & v_{12} & v_{13} & \cdots & f_{1n}\\ v_{21} & v_{22} & v_{23} & \cdots & v_{2n}\\ \vdots & \vdots & \vdots & \ddots & \vdots \\ v_{m1} & v_{m2} & v_{m3} & \cdots & v_{mn} \end{array}\right] \end{array}$$

#### 4.5.4. Calculate the Values for $S_{i}$ and $R_{i}$

VIKOR uses $S_{i}$ as a strategy of maximum utility value and $R_{i}$ as an individual regret value to define and rank the alternatives. These values are needed for the further step of calculation of $Q_{i}$ value. $S_{i}$ and $R_{i}$ values are found based on the Manhattan distance (Equation (6)) and Chebyshev distance (Equation (7)):

(6) $$\begin{array}{c} S_{i}=\sum_{j=1}^{n}w_{j}\frac{f_{j}^{+}-f_{ij}}{f_{j}^{+}-f_{ij}^{-}}=\sum_{j=1}^{n}v_{ij} \end{array}$$

(7) $$\begin{array}{c} R_{i}=\max_{j}w_{j}\frac{f_{j}^{+}-f_{ij}}{f_{j}^{+}-f_{ij}^{-}}=\max_{j}v_{ij} \end{array}$$

#### 4.5.5. Calculate the $Q_{i}$ Values

The aggregation process for $S_{i}$ and $R_{i}$ values is conducted to calculate the $Q_{i}$ values for decision-making. For the case of a possible change in alternative rankings based on $S_{i}$ and $R_{i}$ values, an aggregated measure is utilized. Basically, the best alternative is considered as the alternative with the minimum value of $Q_{i}$, where the alternatives are ranked based on the $Q_{i}$ information. As indicated in Equations (8) and (9), $Q_{i}$ is the integration of normalized values of $S_{i}$ and $R_{i}$, and *v* values as a weighting multiplier.

(8) $$Q_{i}=v\times \frac{S_{i}-S^{+}}{S^{-}-S^{+}}+\left(1-v\right)\times \frac{R_{i}-R^{+}}{R^{-}-R^{+}}$$

where

(9) $$S_{i}^{+}=\min_{i}S_{i}\;,\;S_{i}^{-}=\max_{i}S_{i}\;\mathrm{and}\;R_{i}^{+}=\min_{i}R_{i}\;,\;R_{i}^{-}=\max_{i}R_{i}$$

#### 4.5.6. Analyze the Results

This step is the final step of the VIKOR algorithm. In this step, the controls are performed on whether the results satisfy the given two conditions:

- *Condition 1* deals with the acceptable advantage.
  Assume that $Q(a^{\prime })$ and $Q(a^{\prime \prime })$ are the first- and second-best alternatives, and DQ is 1/(m−1).
  If $Q\left(a^{\prime \prime }\right)-Q\left(a^{\prime }\right)\geq DQ$, it means Condition 1, an acceptable advantage is met.
- *Condition 2* deals with the acceptable stability in decision-making.
  Alternative $a^{\prime }$ must also be the best-ranked by *S* and/or *R*.

If both conditions are met and satisfied, the alternative ranking to the $Q_{i}$ values is said to be true.

## 5. Evaluation

We evaluated our approach by comparing the data transfer performance of the storage option selected by our method against the best guess region (one of the available options within the same geographic region as the data pipeline server) and demonstrated the feasibility of dynamic selection storage options based on four primary user scenarios. A big data pipeline is deployed and tested with all the abovementioned characteristics. Five parameters are used as evaluation criteria to rank different cloud storage options based on the user’s requirements, and algorithms and software tools are developed accordingly. The results from each software tool are then input into the evaluation matrix and used as input for the ranking method. For this article, we considered three cloud storage providers: AWS, Azure, and GCP.

### 5.1. Performance Comparison

**Figure 6:**
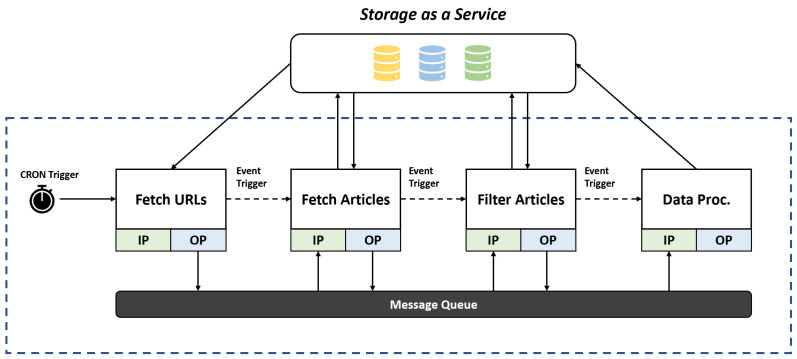
Example data pipeline used in the experiments.

The example pipeline used in the performance evaluation is depicted in Figure 6. The dataset used in the experiments is articles from different online blogs and news websites, each occupying a few kilobytes (KBs) of space on a disk. The use of bigger data chunks is avoided to increase the number of disk operations, which also means less use of computing power. The first step of the data pipeline fetches the URLs from the storage and checks if they are valid. After completing the operation, a flag is set against each URL. The second step downloads only the validated URLs from the storage, then fetches articles from each online source and stores them in the database. The third step downloads the articles from the storage, strips HTML and Javascript tags from the text, and then stores them back in the storage. The fourth step downloads the cleaned text and splits it into smaller data chunks. Since the experiment was to study the role and impact of cloud storage in big data pipelines, to make sure the results are a true representation of the role of StaaS in the performance of a big data pipeline, simple textual data are used so that the least amount of time is spent on actual processing of the data. This way, the execution time of the data pipeline reflects the more precise role of the storage service being used.

**Figure 7:**
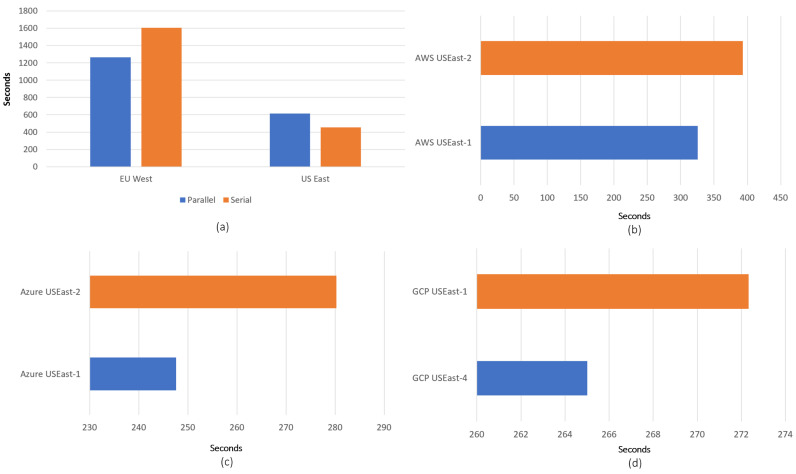
The results of performance experiments: (**a**) Parallel vs. serial execution using the region suggested by our approach for AWS; (**b**–**d**) region pair comparisons in parallel execution for all providers using region suggested by our approach and best guess region.

For the first experiment, a pipeline server is set up in New York (US East, 1 CPU, 2 GB RAM, 50 GB SSD Storage, Ubuntu 20.04) and two storage servers are set up, one in the US East region and another in the EU West region, all using AWS. The dataset used has a size of 4 MB with 3063 variable size data chunks. US East was suggested by our method based only on proximity. The compute steps were run multiple times with both storage services separately in a serial and parallel manner. In serial execution, each step of the data pipeline was run in order, one after the other. In parallel execution, all of the compute steps were run at the same time, in parallel. However, before the execution, it was ensured that the input requirements for each step were met. The results are depicted in Figure 7a, showing the comparison of pipeline execution in a serial vs. parallel manner for both regions. As shown, even the serial execution in the US East region is faster than the parallel execution in the EU West region.

In the second experiment, the data pipeline is deployed on a server located in the US East region. The data pipeline is then integrated with six different storage facilities: two from AWS, two from Azure, and two from GCP. Since the pipeline server is located in the US East region, one can choose between available regions, *us-east-1* to *us-east-4*. Our method suggested the *us-east-4* region instead of the first region, with a sole user weight on the proximity. We set up storage on both region pairs and tested the performance. During the test, a data pipeline with a total of 3601 WRITE operations was carried out, with a total size of 3.2 megabytes (MB). To avoid temporary downtime, the operation was repeated three times. Execution time with the region suggested outranks the best guess region. The results for parallel execution for all three providers are shown in Figure 7b–d.

### 5.2. Cost Comparison

Cloud storage costs might vary widely depending on a company’s needs. Everything from retrieval frequency and storage capacity to network bandwidth affects the cost. We collected and normalized cost data for the three providers in order to be able to compare costs across these providers under different usage scenarios. Mainly, the cost of data storage depends on the amount of data stored, the amount of data transferred through the network, and the number of data transactions. Further, there are multiple storage tiers, each of which has a different pricing structure. Similarly, network usage costs vary depending on whether data are transferred within or outside of the cloud network.

**Figure 8:**
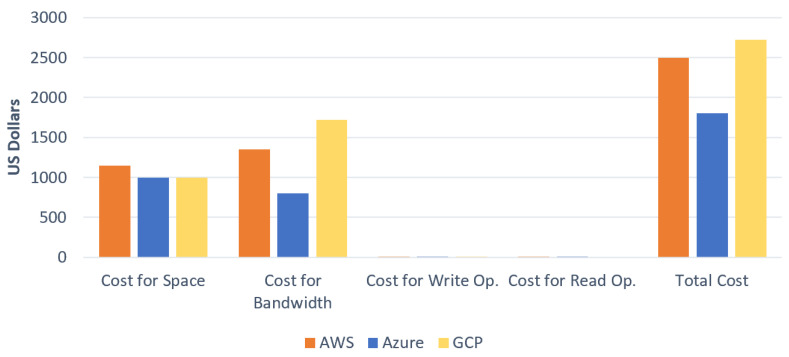
Per-month cost comparison of AWS, Azure, and GCP for 5 TB space, 15 TB bandwidth, 50 thousand write operations, and 50 thousand read operations.

Table 1 gives a fragment of collected data for Azure (EU West) and AWS (EU Stockholm) for the premium storage tier and international data transfer on the network (government clouds are not considered). For example, the cost distribution is shown in Figure 8, given the cost data collected and the following user requirements:

Space = 5000 GB.

Bandwidth = 15,000 GB.

# of WRITE Op. = 50,000.

# of READ Op. = 50,000.

Note that this comparison is meant to be neither conclusive/exhaustive nor an indicator for a particular provider. It is only meant to demonstrate that cost is a complex construct; it can vary considerably depending on the user scenario and requirement, with different results. One also needs to consider cost together with other Q&S attributes [4]. The cost data discussed here are used in the scenarios presented in the following subsection.

### 5.3. User Scenarios

Underlying workload characteristics play an important role in the selection of storage options, as there is often a trade-off between different user requirements. Therefore, we should consider the application portfolio as a whole as well as individually. The following are the user scenarios that are well served by the public cloud.

#### 5.3.1. Scenario 1: Temporary Requirements

As the cloud uses a pay-as-you-go, utility-based pricing model, it is well suited to short-term, transient workloads and projects. Example use cases are proof of concepts, pilots, application testing, product evaluations, training environments, etc. The following user requirements were set up for this scenario:

Space = 1000 GB.

Bandwidth = 15,000 GB.

WRITE Op. = 5000.

READ Op. = 5000.

Table 2 ranks the cloud providers when a user places equal weights for all parameters, that is, 25% for cost, proximity, the impact of encryption, and network performance. Based on these inputs and the momentary conditions, AWS is ranked first by the algorithm. Since the data will be stored for a short period of time, the cost is not the deciding factor here. Instead, in a situation where there would be more frequent read and write operations and higher consumption of network resources, the algorithm provides a fine-tuned compromised solution.

#### 5.3.2. Scenario 2: Highly Variable Workloads

Demand variability comes in two distinct flavors: predictable (seasonal, tidal, cyclical, etc.) and unpredictable. For example, month-end processing, on-season vs. off-season, morning vs. evening, etc. For this scenario, the following requirements were set up:

Space = 2000 GB.

Bandwidth = 25,000 GB.

WRITE Op. = 10,000.

READ Op. = 10,000.

Table 3 ranks the cloud providers when a user places 70% weight on the network performance, 10% on the cost, 10% on the proximity, and 10% on the impact of encryption. In this case, the cost is a secondary factor. Network performance has priority over other factors. In these kinds of situations, it is important that applications work well to avoid any kind of holdup. Network performance is of the utmost importance to make sure there is no bottleneck when there is a high increase in demand, either predictable or unpredictable. Based on these inputs and the momentary conditions, Azure is ranked first by the algorithm.

#### 5.3.3. Scenario 3: High-Security, Low-Scale/Volume Solutions

Although many customers fear the absence of security in the cloud, there are many capabilities within cloud storage to restrict and monitor access to resources. The following requirements were set up for this scenario:

Space = 1000 GB.

Bandwidth = 15,000 GB.

WRITE Op. = 5000.

READ Op. = 5000.

Table 4 ranks the cloud service providers when a user places 80% weight on the impact of encryption, 10% on network performance, and only 5% on cost and proximity. Based on these inputs and the momentary conditions, AWS is ranked first by the algorithm. Since the analysis is carried out for applications with low volume, the cost is a factor on which one can compromise in this situation. However, when data security is of the highest priority, it is important to make sure that it does not impact the overall performance of the application. Our approach ranks the alternatives after analyzing the impact of data security on performance.

#### 5.3.4. Scenario 4: Dormant Workloads

A dormant workload occupies no compute capacity and generates no network traffic, reducing the running costs to just storage. Example use cases are test/development, user acceptance testing, unit, system testing, quality assurance environments, etc. For this scenario, the following requirements were set up:

Space = 5000 GB.

Bandwidth = 1000 GB.

WRITE Op. = 2000.

READ Op. = 2000.

Table 5 ranks the cloud service providers when a user places 70% weight on cost, and 10% each on proximity, network performance, and the impact of encryption. Based on these inputs and the momentary conditions, GCP is ranked first by the algorithm. In this case, data are stored for a long time and are only accessed occasionally. Hence, the user does not have to deal with high network usage costs and data encryption effects. Even low latency is a secondary requirement. The proposed algorithm ranks the alternatives based on the cost of storing data since large amounts of data are stored for archival and backup purposes.

Although the scenarios presented in this section include only a limited number of Q&S attributes, they already demonstrate that storage provider selection is a multifront problem where the preferences of the decision-maker plays an essential role. One also needs to consider trade-offs between different parameters, such as computation vs. storage or network vs. storage [4].

## 6. Conclusions

In this article, we proposed a generic approach for implementing big data pipelines with StaaS integration. This architecture allows hybrid infrastructure, i.e., on-premise processing and on-cloud storage, and local storage temporarily for interstep data input and output. A set of evaluation criteria to rank different cloud storage options based on the user’s requirements was used. Five parameters were selected, and software tools were developed accordingly. The results from each software tool were then input into a matrix (evaluation matrix) and used as input for the ranking method. We tested our approach in terms of data transfer performance and demonstrated its feasibility through four different representative scenarios.

It is important to highlight that the choice of the storage provider is a complex problem that needs to take into account various interrelated parameters depending on the user scenario; even the cost itself is based on complicated and varying price structures. Therefore, the work presented in this article should by no means be considered exhaustive from that perspective. In this respect, regarding future work, firstly, we aim to develop a taxonomy of cost structures and other quality of service parameters playing a role in the selection of storage providers. Then, more parameters can be added to the evaluation matrix. The results of the evaluation matrix could also be compared against actual decision-makers. Secondly, various methods for cost calculation and storage selection based on AI/ML techniques will be explored and compared in order to realize lightweight solutions, which can take various trade-offs into account.

## Figures and Tables

**Table 1:** Example cost data collected for Azure and AWS.

Azure (EU West)		AWS (EU Stockholm)	
Space TB	Price Per GB	Space TB	Price Per GB
0–50	0.02	0–50	0.023
51–500	0.0188	51–500	0.022
51–500	0.018	51–500	0.021
			
Bandwidth IN	Price Per GB	Bandwidth IN	Price Per GB
All	0.00	All	0.00
Bandwidth OUT	Price Per GB	Bandwidth OUT	Price Per GB
0-5	0.0	0–5	0.09
6–15	0.08	0–10	0.09
16–55	0.065	11–50	0.085
56–155	0.06	51–150	0.07
156–500	0.04	>150	0.05
Operations	Price Per 10,000	Operations	Price Per 10,000
Write	0.07	Write	0.05
Read	0.006	Read	0.04

**Table 2:** Scenario 1: Temporary requirements.

Rank	Alternatives	Si	Ri	Qi
1	AWS	0.25	0.15	0
2	GCP	0.28	0.25	0.52
3	Azure	0.75	0.25	1

**Table 3:** Scenario 2: Highly variable workloads.

Rank	Alternatives	Si	Ri	Qi
1	Azure	0.3	0.1	0
2	AWS	0.45	0.42	0.45
3	GCP	0.71	0.70	0

**Table 4:** Scenario 3: High-security, low-scale/volume solutions.

Rank	Alternatives	Si	Ri	Qi
1	AWS	0.08	0.06	0
2	GCP	0.10	0.10	0.04
3	Azure	0.90	0.80	1

**Table 5:** Scenario 4: Dormant workloads.

Rank	Alternatives	Si	Ri	Qi
1	GCP	0.11	0.1	0.01
2	AWS	0.14	0.08	0.02
3	Azure	0.90	0.70	1

## Abbreviations

The following abbreviations are used in this manuscript:

STaaS	Storage-as-a-service
HDD	Hard disk drives
RAID	Redundant array of independent disks
EBS	Elastic block storage
HDFS	Hadoop Distributed File System
